# Resonant Electron Tunneling Induces Isomerization of *π*‐Expanded Oligothiophene Macrocycles in a 2D Crystal

**DOI:** 10.1002/advs.202200557

**Published:** 2022-03-31

**Authors:** José D. Cojal González, Masahiko Iyoda, Jürgen P. Rabe

**Affiliations:** ^1^ Department of Physics and IRIS Adlershof Humboldt‐Universität zu Berlin Newtonstr. 15 Berlin D‐12489 Germany; ^2^ Department of Chemistry Graduate School of Science Tokyo Metropolitan University Hachioji Tokyo 192‐0397 Japan

**Keywords:** molecular electronics, molecular switch, redox isomerization, scanning tunneling microscopy and spectroscopy, solid–liquid interfaces

## Abstract

Macrocyclic oligothiophenes and their *π*‐expanded derivatives constitute versatile building blocks for the design of (supra)molecularly engineered active interfaces, owing to their structural, chemical, and optoelectronic properties. Here, it is demonstrated how resonant tunneling effect induces single molecular isomerization in a 2D crystal, self‐assembled at solid–liquid interfaces under ambient conditions. Monolayers of a series of four *π*‐expanded oligothiophene macrocycles are investigated by means of scanning tunneling microscopy and scanning tunneling spectroscopy (STS) at the interface between their octanoic acid solutions and the basal plane of highly oriented pyrolytic graphite. Current–voltage characteristics confirm the donor‐type character of the macrocycles, with the highest occupied molecular orbital and the lowest unoccupied molecular orbital (LUMO) positions consistent with time‐dependent density functional theory calculations. Cyclic STS measurements show the redox isomerization from *Z,Z*‐8T6A to its isomer *E,E*‐8T6A occurring in the 2D crystal, due to the formation of a negatively charged species when the tunneling current is in resonance with the LUMO of the macrocycle.

## Graphical abstract

Single *Z,Z*‐8T6A molecules forming an ordered 2D crystal are studied using scanning tunneling microscopy and spectroscopy. The current–voltage (*I*–*V)* characteristics of *Z,Z*‐8T6A is measured only when sweeping the bias from negative to positive. Conversely, when sweeping from (high) positive to negative bias, the *I*–*V* characteristics of *E,E*‐8T6A is recorded after the isomerization of *Z,Z*‐8T6A.

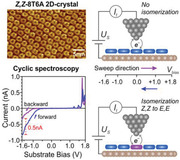

## 1 Introduction

Understanding and controlling molecular transformations on a single‐molecule level constitutes the ultimate level of miniaturization and efficiency toward molecular machines and functional molecular systems.^[^ ^1^, ^2^ ^]^ 2D (supra)molecular self‐assembly is a key strategy toward fabrication of all‐molecular electronic devices that combine functionality, connectivity and high‐density of structures.^[^ ^3^, ^4^, ^5^ ^]^ Tuning the chemical and physical properties of the self‐assembled molecules is therefore crucial for its potential applications.^[^ ^2^, ^3^ ^]^ In this context, the realization of a responsive supramolecular architecture that can be switched in a controllable manner, instead of exhibiting a stochastic response, is desirable for the next generation of molecular devices.^[^ ^6^, ^7^ ^]^ However, the response to external stimuli is critically determined not only by the adequate design of the self‐assembly monolayer (SAM) including its adaptiveness, but as well by molecule–molecule and molecule–substrate interactions.^[^ ^5^, ^8^ ^]^

Scanning tunneling microscopy (STM) provides the capability to investigate the adsorption, assembly, reaction, and electronic properties of molecules at interfaces.^[^ ^3^, ^9^, ^10^, ^11^ ^]^ STM is a unique tool that combines high spatial and energetic resolution with real‐time observation. Furthermore, an STM tip can be used to generate the tunneling conditions required to manipulate and induce transitions at a single molecule level.^[^ ^12^, ^13^, ^14^, ^15^, ^16^ ^]^ By using the electric field in the STM junction, the reversible trans–cis isomerization of azobenzenes derivatives adsorbed on Au(111) was achieved without much perturbation of the 2D crystal.^[^ ^13^ ^]^

At the solid–liquid interface, changes in the substrate bias and voltage pulses applied to the STM tip have induced the reversible switching of conformation and orientation of molecules and complexes physisorbed in 2D crystals.^[^ ^17^, ^18^, ^19^, ^20^, ^21^, ^22^, ^23^ ^]^ Scanning tunneling spectroscopy (STS)^[^ ^24^ ^]^ in an STM‐configuration, allows to create a semiquantitative representation of the local electronic properties of substrates,^[^ ^25^, ^26^ ^]^ molecules,^[^ ^27^ ^]^ and molecular systems.^[^ ^28^, ^29^ ^]^ The measurement of current–voltage (*I*–*V*) characteristics, combining STM/STS techniques, has been used to identify the conductance state of switched molecules,^[^ ^30^, ^31^, ^32^ ^]^ and to elucidate the switching mechanisms involved.^[^ ^33^ ^]^ Even small chemical changes, such as torsional conformational changes,^[^ ^34^ ^]^ may lead to different *I*–*V* characteristics due to the redistribution of the electrons within the system.

Fully conjugated *π*‐expanded oligothiophene macrocycles^[^ ^35^ ^]^ are closely related to redox active oligothiophene macrocycles, which exhibit great potential in optoelectronic applications, organic solar cells, and field‐effect transistors, among many others.^[^ ^36^ ^]^ Moreover, the diastereomers *Z,Z*‐8T6A and *E,E*‐8T6A have been shown to reversible photoisomerize at the solid–liquid interface upon irradiation with light.^[^ ^37^ ^]^

**Scheme 1:**
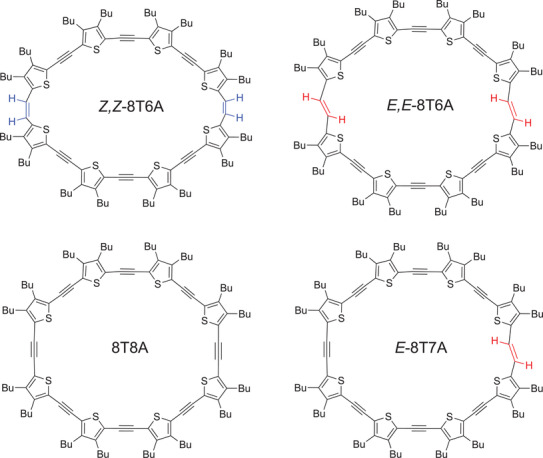
Chemical structures of the macrocycles investigated.

Here, we report on the formation of 2D self‐assembled monolayers of four *π*‐expanded oligothiophene macrocycles (**Scheme** **1**) at the solid–liquid interface. Morphological characterization is obtained from STM images and the electronic characterization from STS measurements. In addition, we show switching in one direction from single molecules of *Z,Z*‐8T6A to their isomers *E,E*‐8T6A after locally increasing the positive substrate bias while measuring *I*–*V* characteristics. Moreover, we provide the proof of principle for resonant electron tunneling‐induced isomerization in‐the‐crystal at a solid–liquid interface.

## 2 Results and Discussion

### 2.1 STM Imaging of Macrocycles at the Solid–Liquid Interface

**Figure 1:**
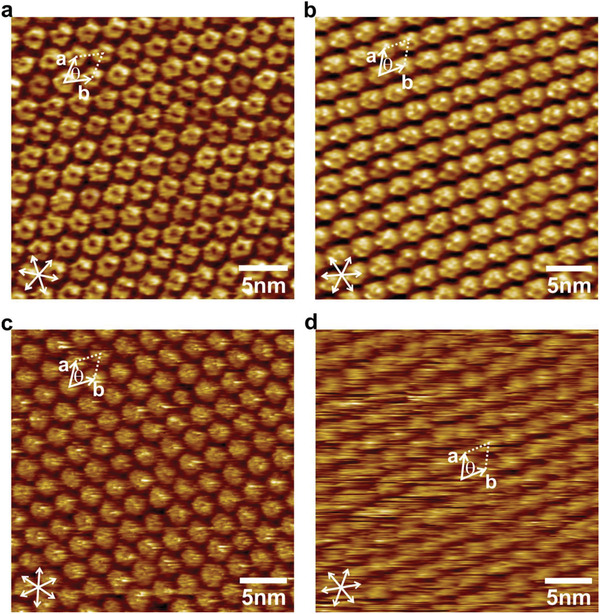
STM height images of the macrocycles adsorbed on HOPG. a) *Z,Z*‐8T6A. b) *E,E*‐8T6A. c) 8T8A. d) *E*‐8T7A. Tunneling parameters: average tunneling current = 90–100 pA, bias voltage = 800–1100 mV (see Table S1 of the Supporting Information for details on the unit cell parameters).

**Figure** **1** shows STM images of the macrocycles at the octanoic acid/graphite interface. The *π*–electron conjugated cores are observed as circular or ellipsoidal high contrast moieties, while due to the large difference in the size of the energy gap,^[^ ^38^ ^]^ the alkyl chains cannot be resolved simultaneously.^[^ ^39^ ^]^ The alkyl chains decorating the macrocycles are actually short, which results in poor interaction of the alkyl groups and the substrate. On the other hand, the stabilization of the self‐assembled monolayer is attributed to the strong *π*–*π* interactions between the fully conjugated electron system and the substrate, expected for this kind of system.^[^ ^35^ ^]^ The contrast of the images is as well related to the rotational symmetry of the conjugated cores: *Z,Z*‐8T6A, *E,E*‐8T6A have a C_2_ and 8T8A has a C_8_ rotational symmetry; while the asymmetry of the conjugated core of *E*‐8T7A explains the poor contrast observed in the STM images.^[^ ^40^ ^]^ While the four macrocycles packed in hexagonal 2D‐patterns with similar unit cell parameters, (2.75–2.92) nm, the isomers *Z,Z*‐8T6A and *E*,*E*‐8T6A differ slightly but significantly, which allows to distinguish them upon, for example, photoisomerization at the solid–liquid interface.^[^ ^38^ ^]^

### 2.2 Redox Isomerization of *Z,Z*‐8T6A in the 2D‐Cystal

According to the resonant tunneling^[^ ^39^, ^41^ ^]^ or orbital mediated tunneling (OMTS)^[^ ^42^ ^]^ model, an asymmetric diode‐like behavior of the *I*–*V* characteristics indicates an asymmetrical alignment of the frontier orbitals with respect to the Fermi level of the closer electrode. Due to the electron donating character of the macrocyclic oligothiophenes,^[^ ^43^, ^44^, ^45^ ^]^ their highest occupied molecular orbital (HOMO) lies closer to the Fermi level of the highly oriented pyrolytic graphite (HOPG) than the lowest unoccupied molecular orbital (LUMO).^[^ ^46^ ^]^

**Figure 2:**
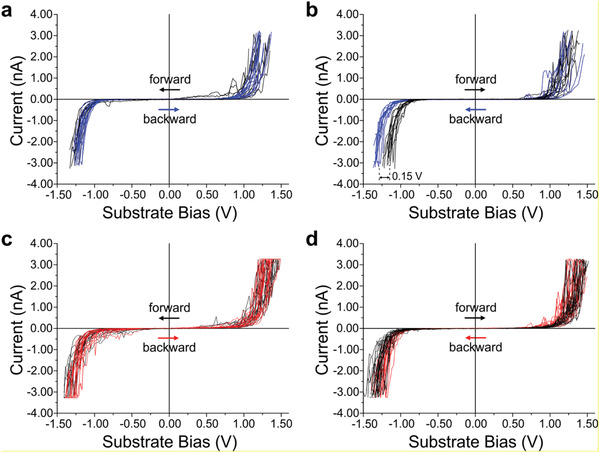
*I*–*V* characteristics across macrocycles a,b) *Z,Z*‐8T6A and c,d) *E,E*‐8T6A, the direction of the sweeping is marked by arrows. (a) 14 measurements and (b) 15 measurements in each direction. (c) 18 measurements and (d) 20 measurements in each direction. Data recorded at substrate bias −900 mV, current setpoint 90 pA. All cyclic scans are from −1.5 to +1.6 V, only the data from −1.3 to +1.5 V is shown.

**Figure** **2**a,c shows the *I*–*V* characteristics through different molecules in SAMs of *Z,Z*‐8T6A and *E,E*‐8T6A. When swept from +1.6 to −1.5 V (substrate bias, *U*_S_) both macrocycles showed equal behavior within the error lines. At positive bias values the current is smaller than at corresponding negative values, which verifies the closer proximity of the HOMO to the Fermi level of the HOPG (closer electrode). On the other hand, in the case of *Z,Z*‐8T6A, when swept from −1.5  to +1.6 V (Figure 2b), the backward trace has a larger tunneling onset voltage (≈0.15 V). In the case of *E*,*E*‐8T6A (Figure 2d), both forward and backward trace show the same aspect, similar to Figure 2c.

The difference between forward and backward trace for *Z*,*Z*‐8T6A (Figure 2a,b) is displayed only at negative substrate values; at positive substrate bias the *I*–*V* characteristics are similar. Moreover, this difference occurs only when started at negative substrate bias, suggesting that at high positive substrate bias a change in the transport characteristics takes place in the molecule. This change could not arise due to different tip‐to‐sample distances because this would also produce a difference in the current at positive bias. In the light of these results, we suggest that the macrocycle *Z,Z*‐8T6A goes through an isomerization process via formation of a charged and/or intermediate state when closely adsorbed to the positively biased HOPG substrate. In this sense, the black curves (forward trace) of Figure 2b represents the actual *I*–*V* characteristics of *Z,Z*‐8T6A, while the blue curves (backward trace) represents the *I*–*V* characteristics of the newly isomerized species *E,E*‐8T6A. A two cycle STS measurement through a single molecule of *Z,Z*‐8T6A confirms the formation of its isomer and the reproducibility of the *I*–*V* characteristics of *E,E*‐8T6A after isomerization (see Figure S1, Supporting Information).

From cyclic voltammetry experiments, it is found that the macrocycle *Z,Z*‐8T6A isomerizes to *E,E*‐8T6A after the formation of a ionic species.^[^ ^38^ ^]^ The formation of ionic species at standard STM conditions cannot be established, since it could compromise the inertness of the solvent. However, in both OMTS^[^ ^42^ ^]^ and superexchange tunneling process^[^ ^47^ ^]^ models, the formation of a short lived (range of microseconds) ionic species is suggested even in the presence of a metallic substrate.

**Figure 3:**
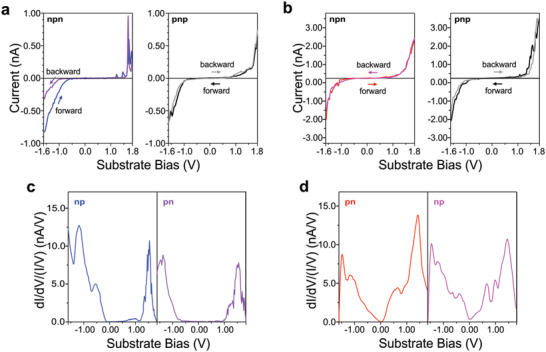
Cyclic STS measurements across a,c) *Z,Z*‐8T6A and b,d) *E,E*‐8T6A. (a) Cyclic scan starting at negative bias (−1.60 V → 1.84 V → −1.60 V, forward blue and backward purple curves, npn) and positive (1.84 V → −1.60 V → 1.84 V, forward black and backward gray curves, pnp). Data recorded when imaging at substrate bias −900 mV and current setpoint 50 pA. (b) Cyclic scan starting at negative bias (−1.60 V → 1.84 V → −1.60 V, forward red and backward magenta curves, npn) and positive (1.84 V → −1.60 V → 1.84 V, forward black and backward gray curves, pnp). Data recorded when imaging at substrate bias −900 mV, current setpoint 75 pA. (c) Numerical derivative (d*I*/d*V*)/(*I*/*V*) of the blue and purple *I*–*V*s in (a). (d) Numerical derivative (d*I*/d*V*)/(*I*/*V*) of the red and magenta *I*–*V*s in (b).

**Figure** **3** shows typical curves of cyclic STS measurements, whose variance deviates less from the average of at least 14 measurements for each case, starting either at negative or positive bias for the isomers *Z,Z*‐8T6A and *E,E*‐8T6A at higher nominal tunneling impedance (larger tip‐to‐sample distance). In cyclic STS experiments, the bias is swept from a maximum negative (positive) value to a maximum positive (negative) and then back to the starting maximum negative (positive) bias. With the help of the numerical derivative of the current, the expression (d*I*/d*V*)/(*I*/*V*) was calculated, which reflects better the local density of states of the molecule.^[^ ^24^, ^48^, ^49^ ^]^ From the blue curve of Figure 3a (−1.6 V → 1.84 V, panel np in Figure 3c) the HOMO and the LUMO of *Z,Z*‐8T6A can be determined at −1.22 and 1.52 V, respectively. Meanwhile, the purple curve in Figure 3a (1.84 V → −1.6 V, panel pn in Figure 3c) is attributed to the newly isomerized macrocycle *E,E*‐8T6A, with LUMO at 1.51 V and HOMO at −1.40 V. From Figure 3b,d, similar frontier orbital positions are obtained for *E,E*‐8T6A at 1.44 V (LUMO) and −1.46 V (HOMO). The sharp features in the blue and purple curves of Figure 3a above 1.4 V are independently obtained several times, and they only appeared when starting from negative bias for the macrocycle *Z,Z*‐8T6A. We consider these features to be an effect of the formation of a charged intermediate species, following the isomerization at positive substrate bias. Furthermore, in all the cyclic experiments, the maximum positive (or negative) bias was kept for 1 ms. This implies that a single isomerization process occurs at a maximum time of 1 ms, with a typical tunneling current of 1 nA at the isomerization bias, giving a yield of 1.6 × 10^−7^ isomerization events per injected electron. This high yield value is strongly enhanced by the resonant tunneling through the unoccupied molecular orbitals of the macrocycle.^[^ ^16^, ^33^ ^]^ No systematic change in the *I*–*V* characteristics was detected below +1.4 V or at negative substrate bias.

STS measurements allow the acquisition of the *I*–*V* characteristics of single molecules forming the 2D hexagonal pattern. In this sense, each probed molecule *Z,Z*‐8T6A got isomerized to *E,E*‐8T6A while being part of the 2D crystal. Since the packing of *Z,Z*‐8T6A and *E,E*‐8T6A is very similar (see Table S1 of the Supporting Information for the unit cell parameters), the isomerization of single molecules of *Z,Z*‐8T6A does not perturb the integrity of the 2D crystal (see Figure S2, Supporting Information). Sometimes, higher contrast moieties were observed in the actual place or nearby (within 5 nm of) the probed molecule (as shown in Figure S2b, Supporting Information). We cannot undoubtedly interpret this observation as an indication of the isomerization event, but we cannot rule this out either.

The macrocycles *Z,Z*‐8T6A and *E,E*‐8T6A are nearly planar and stabilized only by weak interactions with the substrate, thus forming a local capacitive interface, where charges have a longer residence time than in a hypothetically pure ohmic interface with the substrate.^[^ ^50^ ^]^ In addition, the isomerization occurs at positive substrate bias, where the resonant tunneling is assisted by the LUMO of the molecule and the formation of a short lived anionic species is more plausible.^[^ ^51^ ^]^

**Figure 4:**
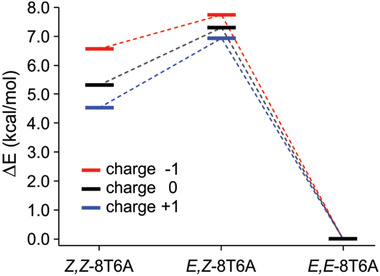
Energy diagram for the redox isomerization process from *Z,Z*‐8T6A to *E,E*‐8T6A in the presence of a homogeneous electric field of 0.15 V Å^−1^. The level shifts of each charged species are given relative to the corresponded energy of the *E,E* isomer.

The photoisomerization between *Z,Z*‐8T6A and *E,E*‐8T6A is two‐step reaction that occurs upon passing through the formation of the unstable *E,Z*‐8T6A.^[^ ^38^ ^]^ **Figure** **4** depicts the potential energy surface (PES) for the transition from *Z,Z*‐8T6A to *E,E*‐8T6A (in their unsubstituted forms), including its transition state *E,Z*‐8T6A. Density functional theory (DFT) calculations were used to determine the energy height for the transition for the neutral, cationic and anionic forms in the presence of a homogeneous electric field of 0.15 V Å^−1^. In the presence of an electric field the energy needed for the anion [*Z,Z*‐8T6A]^−^ to reach the transition state [*E,Z*‐8T6A]^−^ is reduced in comparison to the ground state or the cationic state. This result suggest that the isomerization is a redox reaction involving the formation of the negative species [*Z,Z*‐8T6A]^−^ when the resonant tunneling is assisted by the LUMO of the macrocycles at positive substrate bias. The absence of the (resonant tunneling mediated) reversible isomerization from *E,E*‐8T6A to *Z,Z*‐8T6A at any substrate bias is as well in agreement with the PES results that show a three times higher barrier for the reverse reaction.

**Figure 5:**
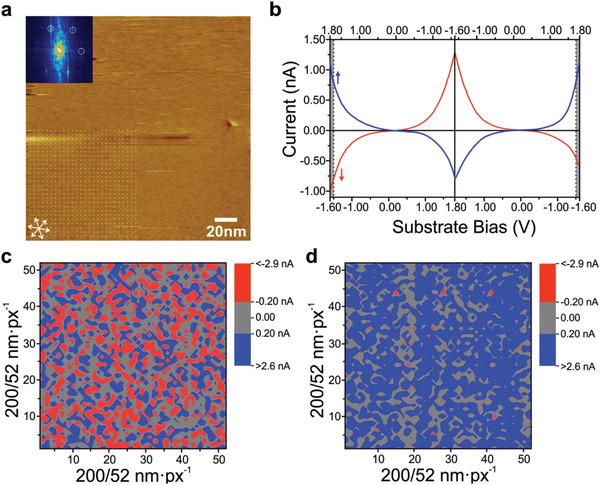
STS difference maps of a 200 nm × 200 nm self‐assembled monolayer of *Z,Z*‐8T6A on HOPG. a) STM height image; 2D fast Fourier transform (FFT) inset highlights the hexagonal pattern. Imaging conditions: substrate bias −900 mV, current setpoint 90 pA. A dotted grid shows part of the STS grid used for the spectroscopy measurements. b) Average cyclic scan from −1.6 to +1.80 V (red curve, npn) and +1.80 to −1.6 V (blue curve, pnp). c) Difference maps between final and starting values for the pnp cyclic scans. d) Difference map between final and starting values for the npn cyclic scans. STS conditions: substrate bias −900 mV, current setpoint 50 pA. The gray areas in (b) are the values used to generate the maps in (c) and (d). The color scale corresponds to one standard deviation above and below the average value.

To validate the statistical significance of the isomerization over large scales, STS difference maps were generated as depicted in **Figure** **5**. These maps were obtained by measuring single *I*–*V* characteristics on a 52 × 52 grid over a 200 nm × 200 nm area. Each bit of the map corresponds to the difference between the tunneling current at starting and final bias values. The average of the (52 × 52 =) 2704 pnp measurements is depicted as a blue curve in Figure 5b, while the average of the 2704 npn measurements is indicated as a red curve. The averaging process smoothened the features given in Figure 3. The presentation of Figure 5b (and Figure S3b, Supporting Information) is intended to highlight the difference of the *I*–*V* characteristics with respect to the direction of the bias sweeping. Figure 5c depicts the difference map for the pnp measurements between 1.7 and 1.8 V for the forward and backward traces (grayed out in Figure 5b). The average distribution of current differences lies around 0 nA, with a mean value of (−0.0037 ± 0.20) nA. This result shows no difference in the forward and backward trace when starting at positive substrate bias. On the other hand, Figure 5d shows the difference map for the npn measurements between −1.5 and −1.6 V. In this case, most of the values lie between 0.024 and 0.44 nA, with a mean value of (0.23 ± 0.20) nA. This last difference map indicates that when the measurement starts at negative substrate bias, the forward trace has consistently larger current values than the backward trace at the largest negative voltage, meaning that for the npn case most of the probed *Z,Z*‐8T6A molecules isomerized to *E,E*‐8T6A.

### 2.3 Frontier Orbitals of Macrocycles

STS measurements were used to determine the frontier orbitals of 8T8A and *E*‐8T7A (see Figures S3 and S4 of the Supporting Information for details) similarly to *Z,Z*‐8T6A and *E,E*‐8T6A.

The energy of the frontier orbitals HOMO and LUMO and the theoretical absorptivity spectra of the macrocycles were determined using time‐dependent DFT (TD‐DFT) calculations at the CAM‐B3LYP/6‐311G(d,p) level. TD‐DFT extends normal (time‐independent) DFT into the time domain, thus allowing more precision in the treatment of electronically excited states. TD‐DFT has been previously used to evaluate electronic transitions in cyclic oligothiophenes^[^ ^52^ ^]^ and complexes of them.^[^ ^53^ ^]^ The theoretical optical gap values were determined from the maximum energy of the theoretical absorptivity, simulated using the major allowed electronic transitions (see Table S2, Supporting Information), and were found to be in good agreement with the experimental results from UV/vis measurements in dichloromethane solutions (see **Table** **1**).

**Table 1:** Energy levels, in eV, of frontier orbitals of macrocycles

Macrocycle	HOMO (DFT)^a)^	HOMO (STS)^b)^	LUMO (DFT)	LUMO (STS)	Optical gap (DFT)	Transport gap (STS)	Optical gap (Sol)^c)^
*Z,Z*‐8T6A	−6.02	−5.7	−2.03	−3.0	2.86	2.7	3.02
*E,E*‐8T6A	−6.19	−5.9	−2.06	−3.0	2.84	2.9	2.80
8T8A	−6.26	−5.9	−2.06	−3.0	2.92	2.9	2.85
*E*‐8T7A	−6.19	−5.7	−1.99	−3.0	2.91	2.7	2.82

^a)^ HOMO and LUMO level of unsubstituted macrocycles estimated at the CAM‐B3LYP/6‐311G(d,p) level.^[^ ^38^ ^]^ Optical gap is the energy of the maximum absorption, simulated from the major allowed transitions determined by TD‐DFT;

^b)^ STS measurements in present study (HOPG work function 4.5 eV^[^ ^58^ ^]^);

^c)^ From optical measurements in solutions of CH_2_Cl_2_ at 25 °C.

Moreover, the values for optical gaps are around 1 eV smaller than the difference E_LUMO_ − E_HOMO_ from TD‐DFT calculations. The optical gap, both experimental and theoretical, corresponds to the energy of the lowest allowed electronic transition(s) via absorption, which in the present case involves a combination of different contributions (see Table S2, Supporting Information). In addition, the band gap measured by STM corresponds to the transport gap, which is the minimum energy necessary to induce a positive charge in the molecule minus the energy gained by adding a negative charge in the same molecule. By definition, this transport gap would be equivalent to the fundamental gap, which can be defined as the difference between the ionization potential and electron affinity. However, in the case of a charged single molecule, the transport gap is typically smaller than the fundamental one, mainly due to the strong polarization of adjacent molecules and/or the substrate, this difference is around 1 eV in *π*‐conjugated molecules,^[^ ^54^ ^]^ consistent with the difference measured for the macrocycles.

Table 1 summarizes the energy levels of the frontier orbitals measured by cyclic voltammetry, estimated from DFT calculations using unsubstituted macrocycles, and from the STS measurements in the present study. The position of the HOMO and LUMO measured by STS is independent of the amount of ethynylene and ethylene groups. Additionally, the position of the LUMO lies at −3.0 eV for all the macrocycles, which is not surprising due to the donor like behavior of the oligothiophene macrocycles.^[^ ^38^ ^]^ TD‐DFT calculations showed a very similar LUMO for all the macrocycles, with a maximum difference of 0.07 eV among them. Meanwhile, the TD‐DFT HOMO values lie closer to the ones measured by STS than the LUMO values, in accordance with the donor character of the macrocycles. Moreover, TD‐DFT results confirm the difference in HOMO values from STS measurements of 0.2 eV between the photoisomers *Z,Z*‐8T6A and *E,E*‐8T6A. This difference allows to distinguish their *I*–*V* characteristics and helped to determine the redox isomerization. It is important to notice as well that the optical gaps (both experimental and theoretical) are mostly within 0.2 eV of the effective gap measured here.

It has to be pointed out that there is a significant difference between the position of the HOMO and LUMO levels measured by STS and the DFT values. Once aromatic molecules are adsorbed on a (semi)metallic substrate like HOPG, shifts in the frontier levels can be induced by interface dipole effects,^[^ ^55^ ^]^ charge transfer from the substrate,^[^ ^56^ ^]^ and variations in the work function of the HOPG.^[^ ^57^, ^58^ ^]^

## 3 Conclusion

A series of four *π*‐expanded oligothiophene macrocycles were self‐assembled in monolayers at solid–liquid interfaces. Despite changes in the symmetry of their cores, a similar hexagonal packing for all macrocycles was obtained. STS measurements allowed us to determine the transport gap of the macrocycles in agreement within 0.2 eV to the optical gap determined by TD‐DFT. Moreover, we have shown the in‐the‐crystal isomerization from *Z,Z*‐8T6A to *E,E*‐8T6A after applying a positive substrate bias, following an anionic route as revealed by the PES from DFT calculations. The changes were followed in situ by detecting conductance hysteresis in the cyclic *I*–*V* characteristics. A statistical analysis over large areas verifies the robustness of the isomerization process. To the best of our knowledge, this study represents the first reported case of single molecular resonant electron tunneling‐induced isomerization in‐the‐crystal at solid–liquid interfaces.

## 4 Experimental Section

### STM Investigation

STM measurements were performed at the interface between HOPG and solutions of *Z,Z*‐8T6A, *E,E*‐8T6A, 8T8A, and *E*‐8T7A (5 × 10^−5^ m) in octanoic acid, employing a home‐built scanning tunneling microscope^[^ ^59^ ^]^ with an Omicron controller. The substrates were glued to a glass holder and electrically contacted using silver paste. STM tips were mechanically cut from a Pt/Ir wire (90/10, 0.25 mm diameter). The STM data were processed by applying a background flattening and the thermal drift was corrected with respect to the known hexagonal HOPG lattice underneath, employing SPIP software (Image Metrology A/S). The HOPG lattice was visualized by lowering the tunneling voltage to 32 mV and raising the tunneling current to 1.4 nA. The white colored axes shown in the STM images of Figure 1 indicate the direction of the main axes of the graphite lattice.

For the STS measurements the STM tip was moved to the position of interest with the feedback on. Consequently, the tip‐to‐substrate distance was kept constant (for each sample) before the feedback loop was interrupted. The feedback loop was then switched off, a bias ramp was swept through and the tunneling current recorded. The starting bias was kept for 2 ms to avoid interference due to faradaic currents. The time between bias steps was 200 µs with an averaged measured time of 50 µs. For the cyclic scans, the middle bias was kept for an additional 1 ms. The *I*–*V* curves displayed in Figure 3 are single measurements whose variance from the corresponding average curve is the minimum. This procedure is done in order to avoid the presence and/or absence of features due to the averaging process.

### Computational Details

DFT calculations were used to determine the PES (Figure 4) and the electronic transitions involving excited states (Table 1). A three‐parameter hybrid exchange functional^[^ ^60^ ^]^ combined with the Lee–Yang–Parr^[^ ^61^ ^]^ correlation functional (B3LYP) was used for geometry optimization and point energy calculations. For TD‐DFT calculations, the hybrid exchange‐correlation functional Coulomb‐attenuating method (CAM‐B3LYP)^[^ ^62^ ^]^ was employed. All the DFT calculation were done using Gaussian09^[^ ^63^ ^]^ program, running on an Debian 7 Linux server in one Intel Core i7‐4770 processor at 3.40 GHz.

### Absorption Spectra

Absorption spectra were recorded at room temperature in CH_2_Cl_2_ solutions contained in 10 mm quartz cuvettes by using a double beam spectrometer (UV‐2101PC, Shimadzu) with blank reference. The solvent was purchased from Aldrich and used without further purification.

### Statistical Analysis

The unit cell parameters of the images in Figure 1 (presented in Table S1, Supporting Information) were determined by the use of at least 10 experimental values (HOPG corrected STM images) from at least three different samples. In Figure 5b, each *I*–*V* curve is the average of 52 × 52 individual measurements. In Figure 5c,d, the color scale corresponds to one standard deviation above and below the average value. The average of the *I*–*V* curves is obtained by taking the normal logarithm of the current to discard inappropriate data which could arise by fluctuations of the tip structure.^[^ ^64^ ^]^ In Figures S3c and S4b (Supporting Information), the error lines are one standard deviation above and below the average value. All plotting and analysis were conducted with Origin 9.0.

## Conflict of Interest

The authors declare no conflict of interest.

## Supporting information

Supporting Information

## Data Availability

The data that support the findings of this study are available from the corresponding author upon reasonable request.
