# Supplementary material for: Resonant Electron Tunneling Induces Isomerization of π‐Expanded Oligothiophene Macrocycles in a 2D Crystal
Source: Adv Sci (Weinh). 2022 Mar 31;9(19):2200557. doi: 10.1002/advs.202200557 (PMC9259718; doi:10.1002/advs.202200557)
Supplement: Supplementary file 1 — Supporting Information [file ADVS-9-2200557-s001.pdf]

ADVANCED SCIENCE
Open Access

Supporting Information

for *Adv. Sci.*, DOI 10.1002/advs.202200557

Resonant Electron Tunneling Induces Isomerization of π-Expanded Oligothiophene Macrocycles in a 2D Crystal

*José D. Cojal González, Masahiko Iyoda and Jürgen P. Rabe**

Supporting Information

Resonant electron tunneling induces isomerization of π-expanded oligothiophene macrocycles in a two-dimensional crystal

*José D. Cojal González, Masahiko Iyoda and Jürgen P. Rabe**

Table S1. Unit cell parameters of self-assembled monolayers of macrocycles in Figure 1 of main text.

Macrocycle	a (nm)	b (nm)	θ (°)
Z,Z-8T6A	(2.75 ± 0.03)	(2.78 ± 0.04)	(61 ± 1)
E,E-8T6A	(2.89 ± 0.02)	(2.92 ± 0.03)	(61 ± 1)
8T8A	(2.75 ± 0.02)	(2.75 ± 0.03)	(60 ± 1)
E-8T7A	(2.77 ± 0.04)	(2.74 ± 0.03)	(61 ± 1)

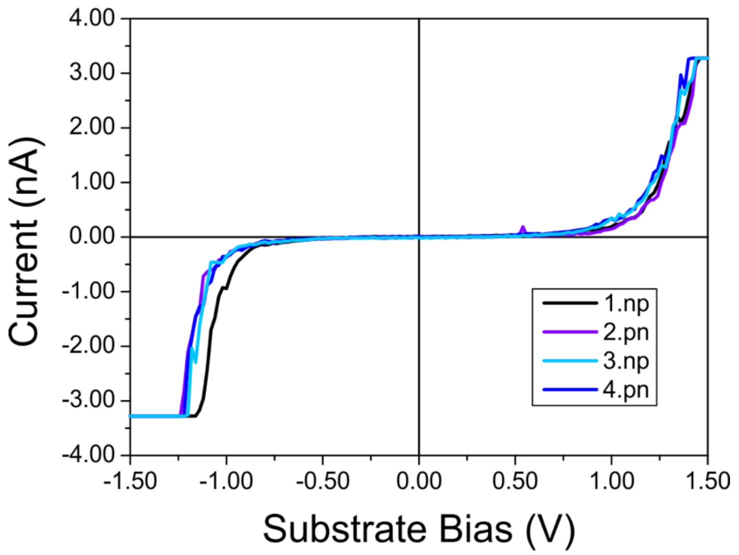

Figure S1. I–V characteristics across *Z,Z*-8T6A corresponding to two cyclic scans from −1.6 V to +1.8 V (1.np), +1.8 V to -1.6 V (2.np), −1.6 V to +1.8 V (3.np) and +1.8 V to -1.6 V (4.np). Only the values from −1.5 to +1.5 V are shown. Data recorded at substrate bias −0.90 V, current setpoint 90 pA.

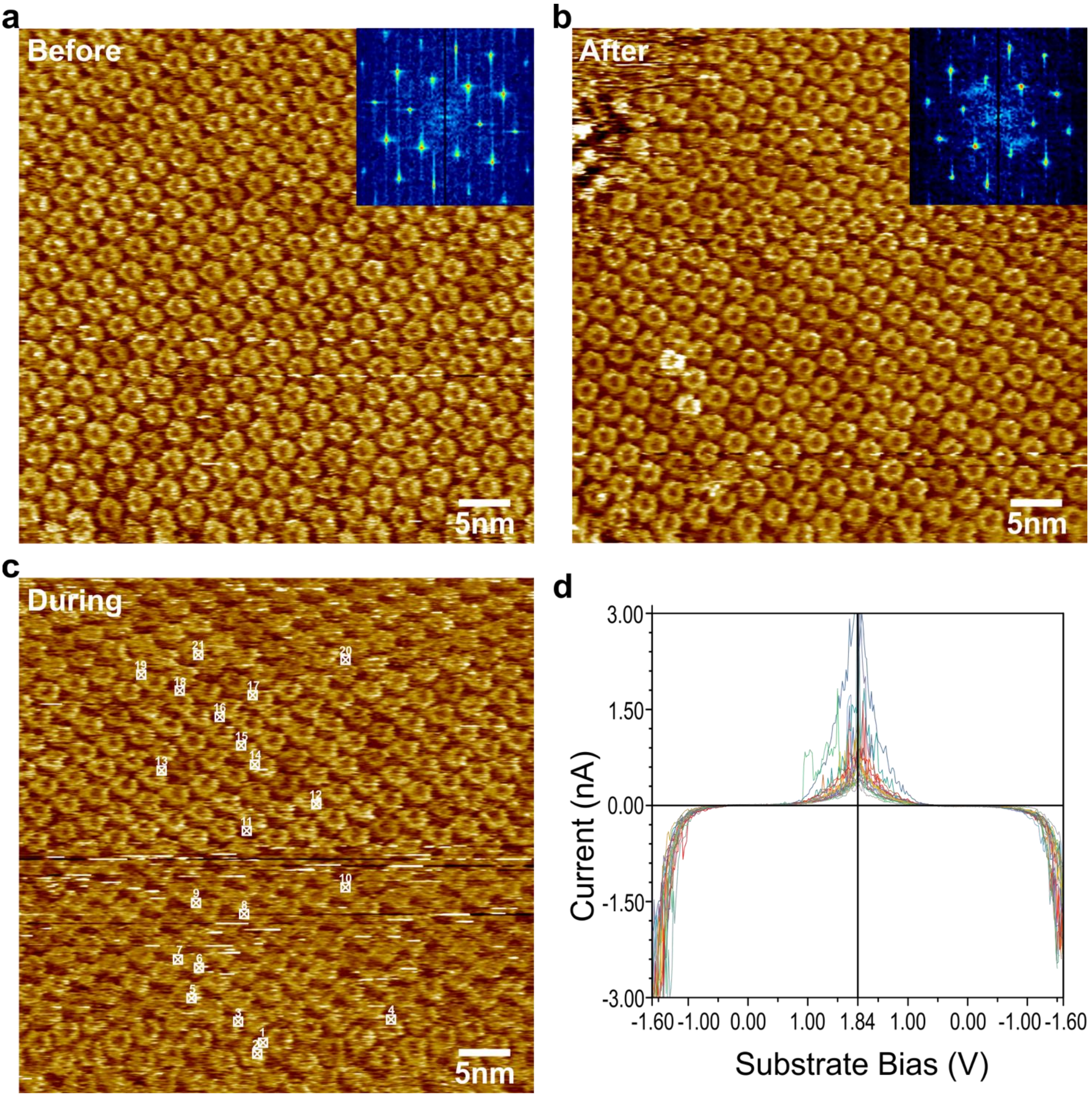

Figure S2. STM height images before (**a**), after (**b**) and during (**c**) cyclic STS measurements in a self-assembled monolayer of *Z*,*Z*-8T6A. (**a**) Fast fourier transform (FFT) shows the crystallinity of the 2D network with unit cell parameters: *a*,*b*=(2.8±0.2), θ=(59±2)°. (**b**) Image taken 6 min after **a**. Unit cell parameters: *a*,*b*=(2.8±0.3), θ=(59±2)°. (**c**) Image taken during 20 I–V characteristics, measured at each white mark. (**d**) Full set of I–V characteristics from **c**. Measurements conditions: substrate bias −900 mV, current setpoint 90 pA. Scan speed 1395nm/s

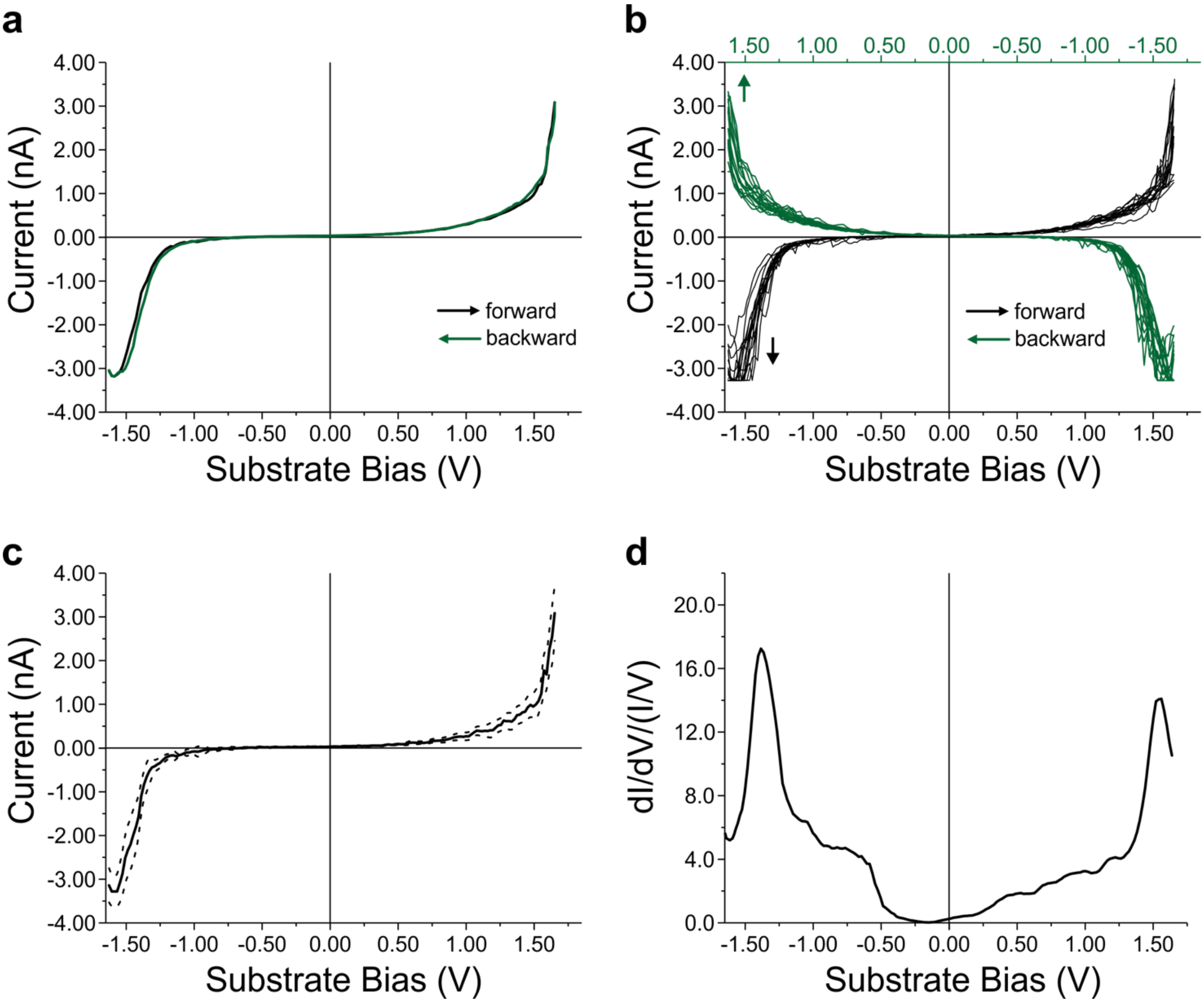

Figure S3. I–V characteristics across the macrocycle 8T8A. (**a**) Forward (black) and back (green) traces are the average of 18 and 22 I–Vs respectively. (**b**) Whole set of data for the average in **a**. (**c**) Single I–V measurement with the error lines of the average of the 18 I–Vs for the forward trace. (**d**) Numerical derivative (dI/dV)/(I/V) of the single I–V in **b**. The data was recorded at substrate bias −900 mV and current setpoint 80 pA.

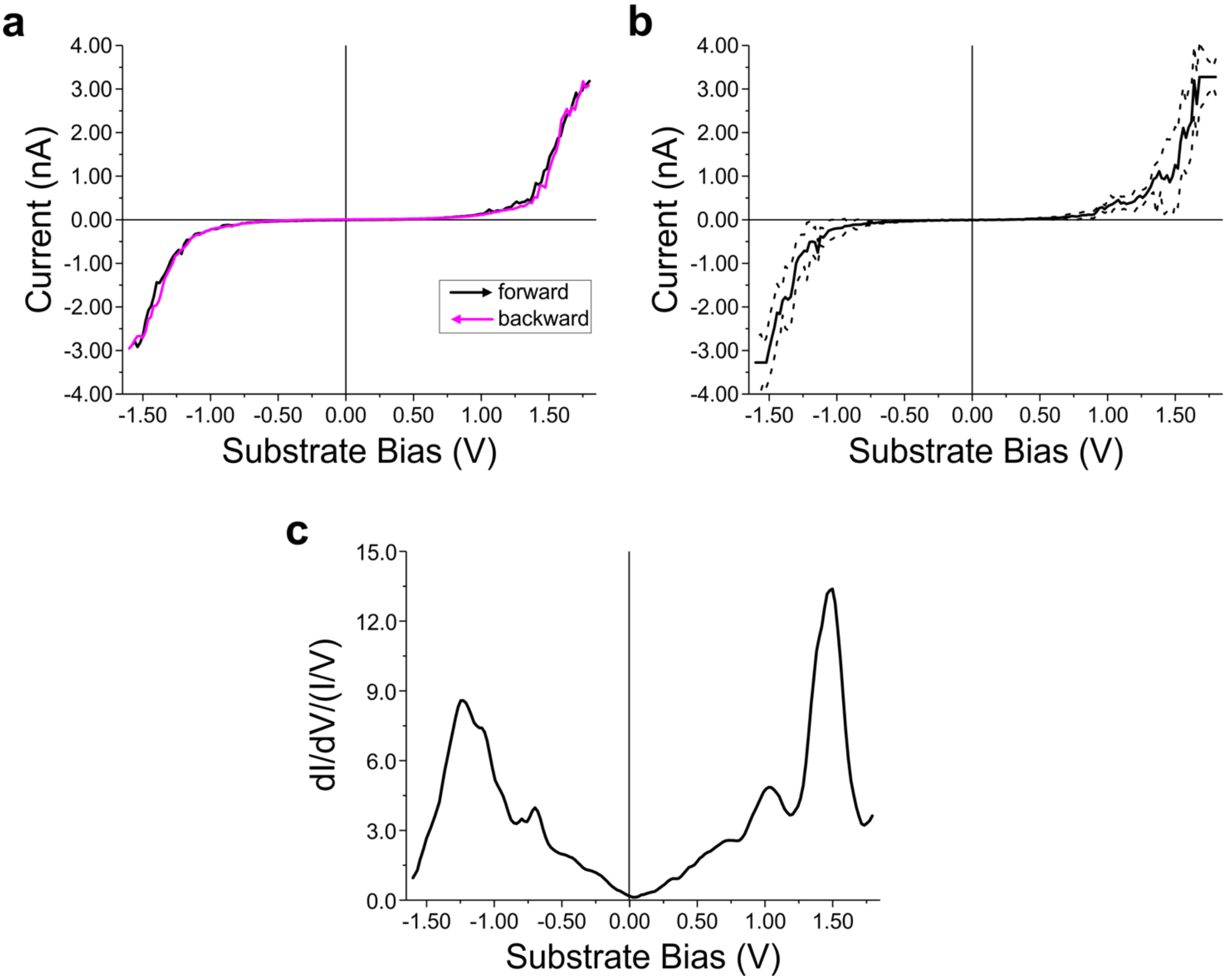

Figure S4. I–V characteristics across the macrocycle *E*-8T7A. (**a**) Forward (black) and back (magenta) traces are the average of 13 I–Vs. (**b**) Single I–V measurement with the error lines of the average of the 13 I–Vs for the forward trace. (**c**) Numerical derivative (dI/dV)/(I/V) of the single I–V in **b**. The data was recorded at substrate bias −950 mV and current setpoint 85 pA.

Table S2. Excited states of 8-mers calculated by TD-DFT at the CAM-B3LYP/6-311G(d,p) level: Calculated excitation energies, wavelengths, oscillator strengths and major contributions of the main allowed transitions in macrocycles.

Macrocycle	Energy (eV)	Wavelength (nm)	Oscillator strength	Significant contributions*
Z,*Z*-8T6A	2.7861<br>2.8992	445.00<br>427.65	1.8222<br>4.3265	H→L+1 (46.6%), H−1→L (46.1%)<br>H→L+2 (47.4%), H−2→L (42.5%)
E,*E*-8T6A	2.7860<br>2.9014	445.00<br>427.32	3.2028<br>2.9715	H→L+1 (47.9%), H−1→L (44.7%)<br>H→L+2 (45.4%), H−2→L (44.6%)
8T8A	2.9225<br>2.9225	424.24<br>424.24	3.0999<br>3.1002	H→L+1 (45.8%), H−1→L (44.2%)<br>H→L+2 (45.8%), H−2→L (44.2%)
E-8T7A	2.3245<br>2.8869<br>2.9368	533.39<br>429.47<br>422.17	0.0299<br>3.1423<br>3.0388	H→L (64.8%), H−1→L+1 (15.7%), H−2→L+2 (14.1%)<br>H→L+1 (49.8%), H−1→L (41.1%)<br>H→L+2 (45.6%), H−2→L (44.5%)

* H and L mean HOMO and LUMO respectively. Only transitions that contribute more than 5% are considered.
